# Does local ambient temperature impact children’s blood pressure? A Chinese National Survey

**DOI:** 10.1186/s12940-016-0119-y

**Published:** 2016-02-16

**Authors:** Qin Li, Yuming Guo, Dong-Mei Wei, Yi Song, Jie-Yun Song, Jun Ma, Hai-Jun Wang

**Affiliations:** Institute of Child and Adolescent Health, School of Public Health, Peking University, Beijing, China; Division of Epidemiology and Biostatistics, School of Public Health, University of Queensland, Brisbane, Australia

**Keywords:** Ambient temperature, Blood pressure, Association, Children

## Abstract

**Background:**

Several studies demonstrated a short-term association between ambient temperature and blood pressure. However, few studies have assessed the long-term effect of ambient temperature on children’s blood pressure. The present study aimed to investigate the association between long-term exposure to local ambient temperature and children’s blood pressure in China.

**Methods:**

We analyzed the systolic (SBP) and diastolic blood pressure (DBP) data of 71,763 children from 2010 Chinese National Survey on Students’ Construction and Health (CHNSCH), and local annual average ambient temperature, relative humidity, air pollutants data from China Meteorological Administration and Ministry of Environment Protection of China. We used generalized additive model (GAM) with non-linear function to examine the effects of ambient temperature on children’s blood pressure.

**Results:**

The results showed that decrease of ambient temperature was negatively associated with increase of both SBP and DBP in Chinese children while adjusting for individual characteristics, socioeconomic conditions, air pollutants and relative humidity. The largest alteration of SBP related to the temperature difference was observed from 20.4 to 9.6 °C, with 9.0 mmHg (95 % CI: 8.4, 9.5) increase in SBP, while the largest alteration of DBP was observed from 21.7 to 10.2 °C, with 6.1 mmHg (95 % CI: 5.6, 6.6) increase in DBP. However, when temperature below 9.6 and 10.2 °C, SBP and DBP started to decrease, which might be caused by the use of heating system in the extreme cold areas.

**Conclusions:**

Public health policy should be improved for protecting children’s cardiovascular health from adverse effects of low temperature. Development of heating system in moderate cold area might be a good solution.

**Electronic supplementary material:**

The online version of this article (doi:10.1186/s12940-016-0119-y) contains supplementary material, which is available to authorized users.

## Background

Numerous previous studies have reported the short-term relationship between temperature and adverse cardiovascular events [1–3]. Raised arterial blood pressure (BP) is a leading cause of this cardiovascular events [4]. Several studies have shown that BP tends to increase with decreasing ambient temperature in both normotensive and hypertensive populations [5–11]. However, most of the above studies focused on the seasonal variation of BP and estimated the effects of temperature on BP among adults or high-risk populations (e.g., old age, prior-cardiovascular disease and end-stage renal disease).

In comparison to adults, children are impacted by temperature difference more seriously. The exposures that influence children’s health begin before conception—reflecting parents’ behavior or exposures—and continue through pregnancy, childhood, and adolescence [12]. Children’s susceptibility could be resulted from many factors such as the lung defense mechanisms against the changes of climatic circumstance which are not fully evolved compared with adults, the continuing process of growth and development of cardiovascular system, less effective heat adaptation capacity and greater surface area to body mass [12–14]. Furthermore, children’s physical behavior, such as greater physical activity, spending more time outdoors, might increase exposure to ambient environment [15, 16]. A better understanding of the adverse impacts of ambient temperature on children’s BP will provide important information for developing public health policy and community and building design. But we only found one study conducted in Germany which analyzed the seasonal variation of blood pressure of German children [17]. No study has investigated the long-term effect of local ambient temperature on children’s blood pressure.

In this study, we used the data of annual ambient temperature in 30 cities of China, and individual data of 71,763 children from the Chinese National Survey on Students’ Construction and Health (CHNSCH) in 2010, the largest Chinese national cross-sectional survey, to analyze the long-term effect of local ambient temperature on children’s blood pressure (BP). The study specifically aimed to identify whether there are the effects of ambient temperature on children’s BP after adjusting for individual characteristics, socioeconomic conditions, relative humidity and air pollutants.

## Methods

### Study population

**Fig. 1:**
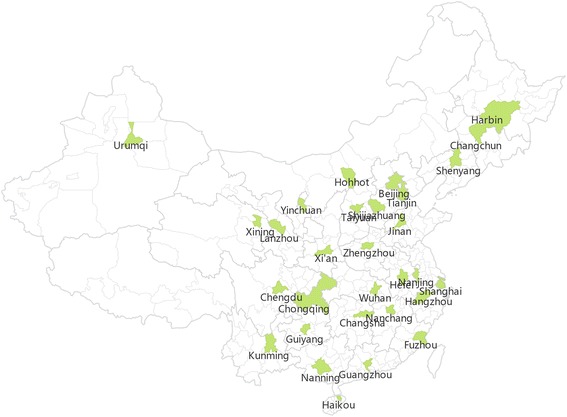
The geographic location of 30 Chinese cities included in this study

We collected the data of children from the 2010 CNSSCH, which was conducted every five years since 1985, jointly launched by the Ministry of Education, the Ministry of Health, the Ministry of Science and Technology, the State of Nation Affairs, and the State Sports General Administration of People’s Republic of China. It is the largest nationally representative sample of school-age children in China. The participants were primary and middle school students aged 7–18 years of Han ethnicity which accounted for 92 % of the total Chinese populations. They were recruited by a stratified multistage sampling procedure from 30 of the 31 mainland provinces, excluding Tibet where Han ethnicity is the minority. The sampling procedure of 2010 CNSSCH has been described in detail elsewhere [18–21]. All eligible participants had lived in the same area for at least one year. They all had medical examination before measurement, to ensure that they had no physical and mental disorders, including respiratory and cardiovascular disorders. In this study, we selected 71,763 subjects aged 7–18 years from 30 cities of china, including 4 municipalities (Beijing, Shanghai, Tianjin, Chongqing) and 26 mainland provincial capital cities (excluding Lhasa, capital of Tibet) (Fig. 1).

### Data collection

#### BP measurements

As recommended by the National High Blood Pressure Education Program Working Group on High Blood Pressure in Children and Adolescents [22], BP readings of the right arm by an auscultation method with a standardized clinical sphygmomanometer was recorded in mmHg. All sphygmomanometer had been calibrated before measurement. A stethoscope was placed over the brachial artery pulse, proximal and medial to the cubital fossa and below the bottom edge of the cuff. The appropriate cuff bladders with different sizes were used to cover at least 40 % of the arm circumference at a point midway between the olecranon and the acromion. BP measurements were taken 5 min after resting. SBP was determined by the onset of the ‘tapping’ Korotkoff sounds (K1) and DBP as the fifth Korotkoff sound (K5). An average of three BP measurements at a single visit was calculated for each child.

#### Anthropometry

Height (cm) and weight (kg) were measured according to the standard procedures in all survey sites [20, 21, 23, 24]. Weight was measured with a standardized scale to the nearest 0.1 kg and height was measured using a wall-mounted stadiometer to the nearest 0.1 cm. Every participant were asked to wear light clothes, to have bare feet, and to stand straight and at ease when being measured. All scales and stadiometers had been calibrated before measurement.

All measurements were conducted from September to November in 2010 by a team of public health professionals in each survey site and they were required to pass a training course for measurements.

#### Physical activity of children

After the anthropometry had been completed, participants at the grade 3 or higher (mostly aged more than 10) were asked to complete a self-administered questionnaire in the classroom with the guidance of trained investigators. The time of physical activity per day, including physical education and extracurricular activities, was collected in the questionnaire as a categorical variable: 1) Less than 30 min per day; 2) Between 30 and 60 min per day; 3) Between 60 and 120 min per day; 4) More than 120 min per day. The physical activity level was dichotomized into physically active and inactive. Physically active children were defined as those who met 1-hour physical activities per day and inactive children were defined as those who didn’t meet, according to WHO recommendations on physical activity for health [25].

#### Data of meteorology and air pollutants

We obtained the annual average ambient temperature (in degrees Celsius) and relative humidity (%) of the 30 cities from 2005 to 2010 from the China Meteorological Administration [26]. Ambient temperature and relative humidity has been continuously measured at fixed sites within each of the cities and published every year. We also acquired the annual average concentration of ambient pollutants such as PM_10_, sulfur dioxide (SO_2_) and nitrogen dioxide (NO_2_) of the 30 cities during the same study period from the Ministry of Environment Protection of China [26].

### Statistical analyses

We examined the association between temperature and the children’s BP by using Generalized Additive Models (GAM). The GAM is proper for identifying the unknown non-linear relationship between dependent variable and explored variables [27]. GAM doesn’t need a priori knowledgeable of the shape of the response curve [28], which is determined by the data itself. In our study, we used children’s SBP or DBP as dependent variable. As BP values were normally distributed, we modelled the association using Gaussian family for BP. We used a spline function for ambient temperature to estimate the relationship between ambient temperature and BP. We used the Akaike’s Information Criterion (AIC) to choose the degree of freedom for the spline. A smaller AIC value indicates the better model [29]. Eventually we adopted 4 of freedom in our model. To control for the cluster effect (e.g., economic status), we modelled school as a categorical variable. As children’s age, gender, height and weight are associated with BP, we controlled for these variables in all analyses. Considering the economic development could impact children’s BP [30], we also controlled for the Gross Domestic Product (GDP) per capital of each city in our models. To adjust for the effects of air pollutants and humidity, we added the annual average concentration of PM_10_, NO_2_, SO_2_ and the relative humidity to the models. As children’s physical activity may be associated to BP [31], we also conducted subgroup analyses in children who had physical activity data. Considering the children have been growing through childhood and puberty periods, we also conducted stratification analyses in different age group to identify the associations. Sensitivity analyses were performed using the data of ambient temperature during the previous 5 years (2005–2009) and average ambient temperature of 2005–2010 to perform the same analyses.

We used software R, version 3.1.0, the “ggplot2” and “maptools” package to produce a map showing geographic location of the 30 cities in China. Map files of boundaries of China, provinces, municipalities and provincial capital cities were obtained from National Fundamental Geographic Information System of China.

All analyses were performed using the statistical software R, version 3.1.0 [32]. The “mgcv” package was used to perform GAM analyses [28]. Results are presented as the estimated changes in SBP/DBP associated with every 1 °C decrease of ambient temperature, with the 95 % confidence interval (95 % CI). A *p* value of <0.05 for 2-sided test was considered as statistically significant.

## Results

There were 71,763 children in this study, with the total sample of each city from 2348 to 2400, including about 100 subjects in each gender-age subgroup. The proportion of female was from 49 to 50 % and the mean age was from 12.4 to 12.5 years in each city. Figure 1 shows geographic location of the 30 cities in this study, being very disperse and representative of different parts of China. There was an obvious geography variation in the annual average temperature among different cities in 2010, ranging from 4.5 °C in Harbin to 24.6 °C in Haikou. The annual average relative humility in 2010 also varied substantially among the 30 cities, ranging from 51 % of Yinchuan and Beijing to 81 % of Haikou (Table 1).

**Table 1:** General characteristics of subjects in 30 cities of China

	Total	Female	Age (year)		Temperature	Humidity
	number	(%)	Mean	SD	(°C)	(%)
Haikou	2378	50.0	12.5	3.5	24.6	81
Guangzhou	2400	50.0	12.5	3.5	22.5	73
Nanning	2372	50.0	12.5	3.4	21.8	77
Fuzhou	2398	50.0	12.5	3.5	20.4	74
Chongqing	2400	50.0	12.5	3.5	18.6	78
Nanchang	2392	50.0	12.5	3.4	18.5	73
Changsha	2398	50.0	12.5	3.5	18.2	74
Hangzhou	2394	49.9	12.5	3.4	17.4	72
Shanghai	2400	50.0	12.5	3.5	17.2	69
Kunming	2400	50.0	12.5	3.5	16.7	66
Wuhan	2399	50.0	12.5	3.5	16.6	77
Hefei	2400	50.0	12.5	3.5	16.4	72
Nanjing	2400	50.0	12.5	3.5	16.2	71
Chengdu	2400	50.0	12.5	3.5	16.0	79
Zhengzhou	2400	50.0	12.5	3.5	15.6	56
Guiyang	2400	50.0	12.5	3.5	14.6	77
Xi’an	2398	50.0	12.5	3.5	14.6	62
Jinan	2377	50.2	12.5	3.5	14.3	54
Shijiazhuang	2374	50.3	12.5	3.4	14.0	55
Beijing	2400	50.0	12.5	3.5	12.6	51
Tianjin	2400	50.0	12.5	3.5	12.2	59
Taiyuan	2400	50.0	12.5	3.5	11.3	52
Yinchuan	2348	50.9	12.4	3.4	10.3	51
Lanzhou	2400	50.0	12.5	3.5	7.9	56
Hohhot	2400	50.0	12.5	3.5	7.6	46
Urumqi	2399	50.0	12.5	3.5	7.4	56
Shenyang	2387	49.9	12.5	3.4	7.2	71
Xining	2369	50.6	12.4	3.4	6.4	54
Changchun	2396	50.1	12.5	3.5	5.2	66
Harbin	2389	49.9	12.5	3.4	4.5	70

*SD* standard deviation, Temperature was the annual average temperature of 2010, Humidity was the annual average relative humidity of 2010

The mean SBP of all 71,763 children was 104.57 mmHg with standard deviation of 12.25 mmHg and the mean SBP increased from 96.32 mmHg at age 7 to 110.59 mmHg at age 18. For DBP, the mean value was 64.28 mmHg with standard deviation of 9.88 mmHg and the mean DBP increased from 59.32 mmHg at age 7 to 68.07 mmHg at age 18 (Table 2).

**Table 2:** Mean SBP/DBP of participates in each age groups

Age	SBP (mmHg)		DBP (mmHg)	
	Mean	S.D	Mean	S.D
7	96.32	10.82	59.32	10.16
8	97.97	10.51	60.63	9.60
9	99.12	11.07	61.69	9.93
10	101.43	11.00	63.19	9.53
11	103.47	10.98	64.20	9.65
12	104.54	11.21	63.70	9.54
13	105.83	11.21	64.11	9.72
14	107.70	11.44	65.51	9.23
15	108.52	11.41	66.19	9.31
16	109.18	11.65	67.12	9.04
17	110.23	12.21	67.75	9.11
18	110.59	12.01	68.07	9.31
Total	104.57	12.25	64.28	9.88

*SD* standard deviation

**Fig. 2:**
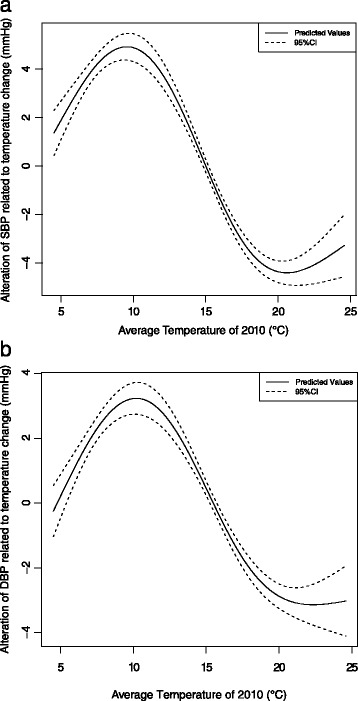
**a** The alteration of children’s SBP related to temperature difference. **b** The alteration of children’s DBP related to temperature difference. *(Generalized additive model, adjusting for effect of school, age, height, weight of each child, average relative humidity of 2010 and GDP per capital of 2010)*

Figure 2a and b showed the association between annual average ambient temperature and children’s SBP for all participants adjusting for relative humidity, GDP per capital and anthropometry (age, gender, height and weight). Low ambient temperature resulted in increase of children’s SBP within certain temperature range (from 20.6 to 9.5 °C) (Fig. 2a). When ambient temperature was lower than 9.5 °C, lower temperature resulted in decrease in SBP and when ambient temperature was higher than 20.6 °C, higher temperature resulted in increase in SBP. Consistently, low ambient temperature resulted in increase of children’s DBP within certain temperature range (from 22.3 to 10.2 °C) (Fig. 2b). When ambient temperature was lower than 10.2 °C, lower temperature resulted in decrease in DBP and when ambient temperature was higher than 22.3 °C, higher temperature resulted in increase in DBP).

**Fig. 3:**
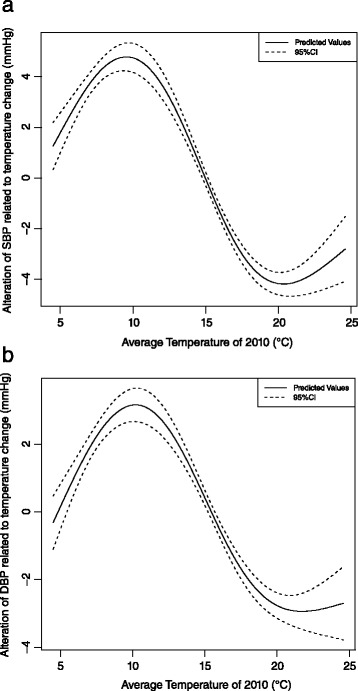
**a** The alteration of children’s SBP related to temperature difference. **b** The alteration of children’s DBP related to temperature difference. *(Generalized additive model, adjusting for effect of school, age, gender, height, weight of each child, concentration of PM*_*10*_ *, NO*_*2*_ *and SO*_*2*_ *of 2010, average relative humidity of 2010 and GDP per capital of 2010)*

When we finally adjusted air pollutants (PM_10_, NO_2_ and SO_2_) in above models, the associations didn’t changed. Low ambient temperature resulted in increase of children’s SBP within certain temperature range (from 20.4 to 9.6 °C) (Fig. 3a). Children’ SBP value achieved the maximum at 9.6 °C (When ambient temperature was lower than 9.6 °C, lower temperature resulted in decrease in SBP) and SBP value achieved minimum at 20.4 °C (When ambient temperature was higher than 20.4 °C, higher temperature resulted in increase in SBP). Consistently, low ambient temperature resulted in increase of children’s DBP within certain temperature range (from 21.7 to 10.2 °C) (Fig. 3b). Children’ DBP value achieved the maximum at 10.2 °C (When ambient temperature was lower than 10.2 °C, lower temperature resulted in decrease in DBP) and DBP value achieved minimum at 21.7 °C (When ambient temperature was higher than 21.7 °C, higher temperature resulted in increase in DBP).

**Fig. 4:**
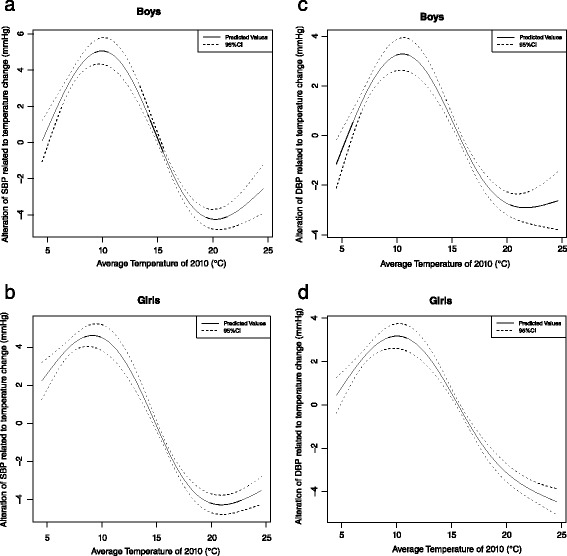
**a** The change of SBP related to temperature difference among boys. **b** The change of DBP related to temperature difference among girls. **c** The change of SBP related to temperature difference among boys. **d** The change of DBP related to temperature difference among girls. *(Generalized additive model, adjusting for effect of school, age, height, weight of each child, concentration of PM*_*10*_ *, NO*_*2*_ *and SO*_*2*_ *of 2010, average relative humidity of 2010 and GDP per capital of 2010)*

Table 3 showed that the largest alteration of SBP related to the temperature difference from 20.4 to 9.6 °C reached 9.0 mmHg (95 % CI: 8.4, 9.5). Between the hottest and coldest area, the alteration of SBP related to the temperature difference from 24.6 to 4.5 °C was 4.1 mmHg (95 % CI: 2.8, 5.3). Besides, the largest alteration of DBP related to the temperature difference from 21.7 to 10.2 °C reached 6.1 mmHg (95 % CI: 5.6, 6.6). Between the hottest and coldest area, the alteration of DBP related to the temperature difference from 24.6 to 4.5 °C was 2.4 mmHg (95 % CI: 1.3, 3.5). In addition, similar trend for both SBP and DBP were found in both boys and girls (Fig. 4a, b, c and d).

**Table 3:** Change in SBP associated with temperature change^a^

Terms	All	Girls	Boys
SBP			
High threshold value^b^	110.3 (109.8, 110.9)	108.9 (108.3, 109.5)	111.5 (110.8, 112.3)
Low threshold value^b^	101.8 (101.3, 102.3)	100.0 (99.5, 100.5)	102.2 (101.7, 102.8)
High threshold VS low threshold	9.0 (8.4, 9.5)	8.9 (8.3, 9.5)	9.3 (8.6, 10.0)
Lowest temperature VS Highest temperature^c^	4.1 (2.8, 5.3)	5.8 (5.0, 6.5)	2.6 (1.3, 4.0)
DBP			
High threshold value^b^	68.1 (67.6, 68.6)	68.1 (67.5, 68.6)	68.1 (67.4, 68.7)
Low threshold value^b^	62.0 (61.4, 62.5)	60.4 (60.0, 61.0)	61.9 (61.3, 62.5)
High threshold VS low threshold	6.1 (5.6, 6.6)	7.6 (7.1, 8.2)	6.2 (5.5, 6.9)
Lowest temperature VS Highest temperature^c^	2.4 (1.3, 3.5)	4.9 (4.3, 5.5)	1.5 (0.3, 2.6)

^a^The values were extracted from Figs. 2a, b and 3a, b, c and d, reporting as mean (95 % CI) in mmHg

^b^When temperature was 9.6/10.2 °C, the SBP/DBP value reaching highest and named high threshold value. When temperature was 20.4/21.7 °C, the SBP/DBP value reaching lowest and named low threshold value

^c^In our data, 24.6 °C is the hottest temperature and 4.5 °C is the lowest temperature

**Fig. 5:**
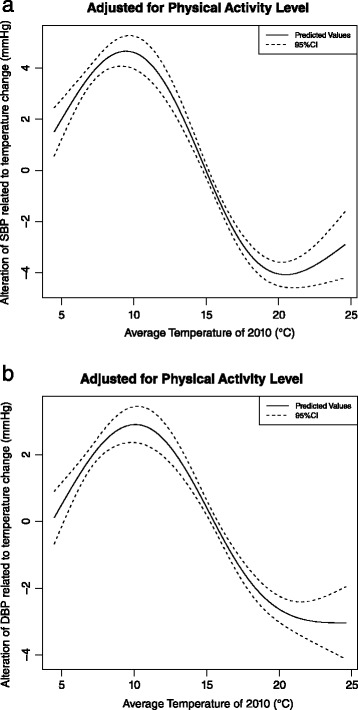
**a** The change of SBP related to temperature difference. **b** The change of DBP related to temperature difference. *(Generalized additive model, subgroup analysis, adjusting for effect of school, age, height, weight, physical activity level of each child, concentration of PM*_*10*_ *, NO*_*2*_ *and SO*_*2*_ *of 2010, average relative humidity of 2010 and GDP per capital of 2010)*

When we conducted subgroup analysis by controlling the physical activity level as a co-variable in our model, the associations also didn’t change, and the effect of physical activity level on SBP and DBP was not significant (*p* = 0.316). (Fig. 5a, b).

The associations between ambient temperature and children’s SBP stratified by the age group were shown in Additional file 1: Figures S1 to S6. We also found that low ambient temperature resulted in decrease of children’s SBP and DBP within certain temperature range in all age groups. The largest alteration of SBP related to the temperature difference reached from 11.1 (95 % CI: 10.2, 11.9) to 23.2 (95 % CI: 22.3, 24.2) mmHg. The largest alteration of DBP related to the temperature difference reached from 7.2 (95 % CI: 6.5, 7.9) to 14.0 (95 % CI: 13.3, 14.8) mmHg.

Sensitivity analyses showed that the effect estimates were very similar to the above findings when we used the data of ambient temperature during the previous 5 years (2005–2009). We also found consistent results when we used the mean temperature from 2005 to 2010. (See Additional file 1: Figures S7 to S12).

## Discussion

To our knowledge, it is the first study to examine the long-term effect of local ambient temperature on children’s blood pressure by using a national data of China. By examining the largest representative sample of school-age children in 30 cities of China (*N* = 71,763), we found that decrease in ambient temperature was associated with increase in both SBP and DBP of children within certain temperature range. The largest increase of SBP could reach 9.0 mmHg (95 % CI: 8.4, 9.5) and the largest increase of DBP could reach 6.1 mmHg (95 % CI: 5.6, 6.6). In addition, children living at extreme hot areas (annual ambient temperature higher than 20 °C) have higher BP than those living at areas with annual temperature of 20 °C.

Several studies have provided evidence about seasonal variation of blood pressure and the effects of ambient temperature on blood pressure in adults or high-risk populations. A study conducted in three French cities by Alpérovitch et al. [5] reported that in 65 years or older people (*N* = 9686), mean SBP in winter was 5.0 mmHg higher than it in summer. They also found SBP increased with decreasing temperature, with an 8.0 mmHg increase between the highest (>21.2 °C) and the lowest (<7.9 °C) temperature quintile. For DBP, they found a 2.9 mmHg increase between the highest and the lowest temperature quintile. Yang et al. [8] analyzed the data on 23,000 individuals with prior-cardiovascular disease and found that mean SBP was significantly higher in winter than in summer and each 10 °C lower ambient temperature was associated with 6.2 mmHg higher SBP. For children, only one study conducted in Germany reported that compared to summer, children’ mean SBP/DBP was increased 4.45/2.42 mmHg during the winter. They also found that between −0.39 and 18.1 °C, with each 1 °C increase in average outdoor temperature, the systolic blood pressure fell by 0.12 mmHg [17].

However, most of the previous studies estimated the effect of temperature on BP by using the seasonal temperature alterations in the same area. In our study, we use the local annul average ambient temperature as exposure and found decrease in ambient temperature was associated with increase in both SBP and DBP of children within certain temperature range in 30 cities of China, which may provide diverse evidence for improving public health policy in cold areas. As previous study had reported, each 10 mmHg increase of SBP was related to 21 % higher risk of CVD mortality [8], our findings suggested temperature should be considered in early prevention of cardiovascular disorders. In view of the severe situation of China’s air pollution and climate change [33], we should also pay more attention to the effects of high level of air pollution and low ambient temperature in children’s cardiovascular health.

In addition, we also found extreme higher temperature was associated with increases in SBP/DBP. When ambient temperature was higher than 20.4/21.7 °C, higher temperature resulted in increase in SBP/DBP. A possible reason could be that in hot areas children are more likely to stay inside buildings, and this might increase the exposure to indoor air pollution (e.g. carbon dioxide, total volatile organic compounds, PM2.5), which would increase the BP level [34]. Besides, it is possible that in hot areas children are more likely to stay inside buildings with air conditioners, and this could dilute the effect of ambient temperature (because indoor cool temperature increases BP).

It is interesting that the association between ambient temperature and SBP/DBP was in reverse when ambient temperature was even colder. When ambient temperature was lower than 9.6/10.2 °C, lower temperature resulted in decrease in SBP/DBP. This might be caused by the use of heating system in north areas (extreme cold) of China, but no heating systems in moderate cold areas [8].

We found consistent associations between ambient temperature and both SBP or DBP before and after adjusted the effect of air pollutants (PM_10_, NO_2_ and SO_2_) in our models. In addition, the largest alteration of SBP related to the temperature difference reached 9.3 mmHg in the model without adjustment for air pollutants compared to 9.0 mmHg in the full model, and the largest alteration of DBP related to the temperature difference reached 6.4 mmHg compared to 6.1 mmHg in the full model. Although air pollution is a major environmental issue in China and it could have a strong impact on children’s health including BP, our results indicated that temperature had independent impact on children’s SBP/DBP. Considering the physical activity level will affect children’s SBP/DBP [31], we also estimated the association between ambient temperature and SBP/DBP by added children’ physical activity level as a co-variable in our models. The association was very similar to the one without physical activity level. It also indicated that temperature had impact on children’s SBP/DBP independently of physical activity.

The associations between ambient temperature and children’s SBP/DBP stratified by the age group showed consistent results. The largest alteration of SBP/DBP related to the temperature difference were various, which could be resulted from the different abilities to adapt to the environmental exposures and the different behavior pattern of each period of child’s growth and learning stages. Public health policy should be developed for protecting children in each age group from the adverse effects of temperature.

There are several mechanisms for increase of blood pressure related to the decrease of temperature, among which activation of the sympathetic nervous system accompanied by secretion of catecholamine and other substances involved in heat production play major roles [35]. Alterations in skin vasomotor tone resulting in a marked increase in vascular peripheral resistances, increase in peripheral vasoconstriction. It can decrease sweating and, therefore, salt loss, both of which would increase blood pressure [36–39]. Another study reported that low temperature is associated with an increase in blood viscosity, levels of red blood cell count [40], which may also influence the blood pressure.

The limitation of this study was that the information on ambient temperature, relative humidity, and air pollution was from fixed monitoring sites of each city, rather than individual exposure. However, ambient temperature is the most frequently used as a proxy of exposure in researches as it is readily available and covers wide area such as a city [41]. Previous researches had also found that average temperature is good at estimating city-wide associations between temperature and mortality because it is strongly correlated within each city [42]. Additionally, as an ecological study, we could not establish the temporal order of cause and effect because of the cross-sectional study design.

Our study has several strengths. First, this is the first national study to examine the long-term effect of local ambient temperature on BP of school children in China. It included the large number of children (*N* = 71,763) from 30 cities of China, having great representativeness of Chinese children. The meteorological environment of the 30 cities included tropic, temperate and frigid climate and the range of annual ambient temperature were from 4.5 to 24.6 °C, which is wider than previous studies [5, 7, 17]. Second, we used GAM and smooth function to estimate the relationship between ambient temperature and BP, and adjusted the socioeconomic status, physical activity level and air pollutants in our model, which had reasonability to identity the association between ambient temperature and BP. After adjusting for air pollutants, we find that the effects of ambient temperature on children’ BP were still significant, which means the effects was independent of air pollutants.

## Conclusion

In conclusion, we found that low ambient temperature was significantly associated with increase of blood pressure in Chinese school children. Public health policy should be developed for protecting children from the adverse effects of temperature on their cardiovascular health during growth and development. Improvement of building design (e.g., installing heating system in moderate cold areas and air conditioners in classrooms for extreme hot areas) might be useful to reduce temperature impacts on children’s cardiac function.

### Ethics approval

The study project was approved by the Medical Research Ethics Committee of the University of Queensland (#2011001199).

## Additional file

Additional file 1:
**Sensitivity analyses by used the data of ambient temperature during the previous 5 years (2005–2009) and  the mean temperature from 2005 to 2010.** (DOCX 1866 kb)
