# Supplementary material for: Does local ambient temperature impact children’s blood pressure? A Chinese National Survey
Source: Environ Health. 2016 Feb 16;15:21. doi: 10.1186/s12940-016-0119-y (PMC4754880; doi:10.1186/s12940-016-0119-y)
Supplement: Additional file 1: — Sensitivity analyses by used the data of ambient temperature during the previous 5 years (2005–2009) and the mean temperature from 2005 to 2010. (DOCX 1866 kb) [file 12940_2016_119_MOESM1_ESM.docx]

Supplement

**
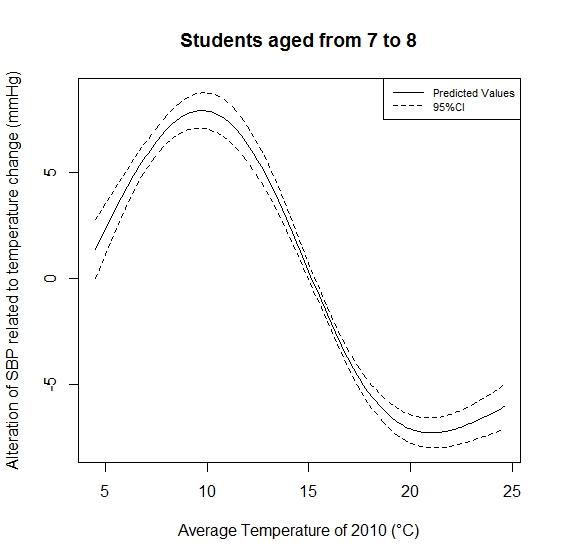
**
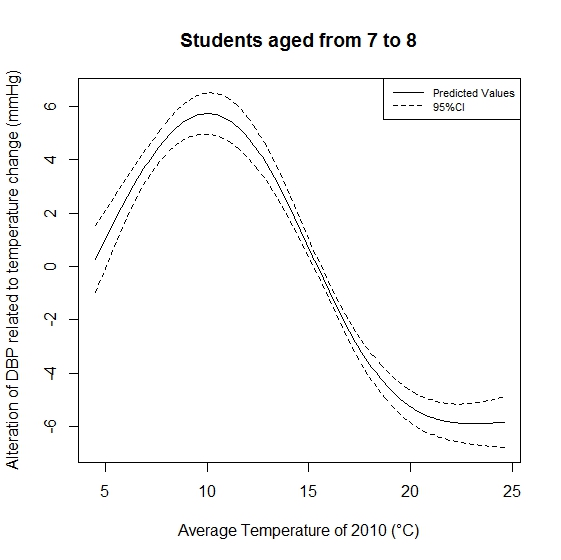


**Figure S1. The alteration of children’s SBP/DBP related to temperature difference**

**(7-8 years old children)**

(*Generalized additive model, adjusting for effect of school, gender, height, weight of each child, concentration of PM_10_, NO_2_ and SO_2_ of 2010, average relative humidity of 2010 and GDP per capital of 2010*)

**
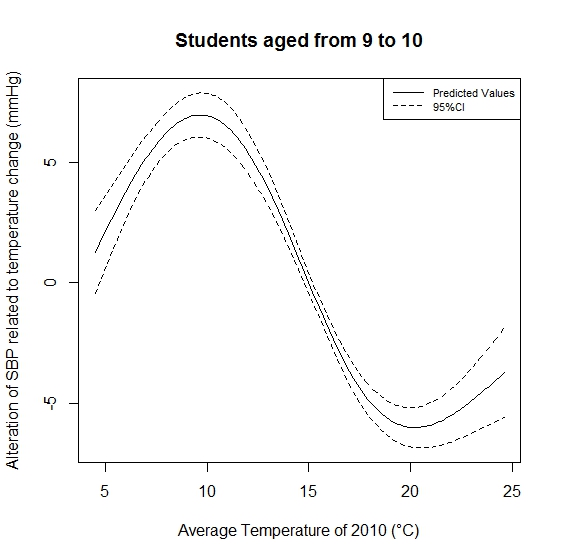
**
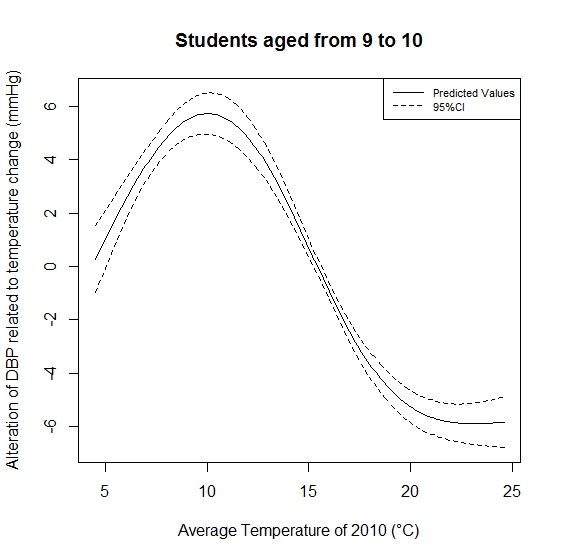


**Figure S2. The alteration of children’s SBP/DBP related to temperature difference**

**(9-10 years old children)**

(*Generalized additive model, adjusting for effect of school, gender, height, weight of each child, concentration of PM_10_, NO_2_ and SO_2_ of 2010, average relative humidity of 2010 and GDP per capital of 2010*)

**
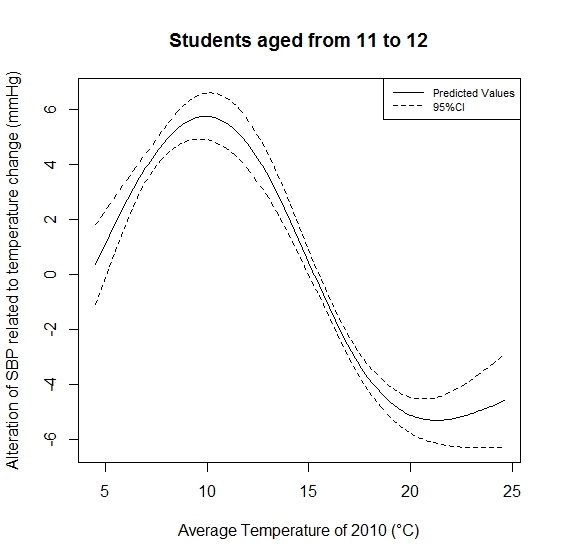
**
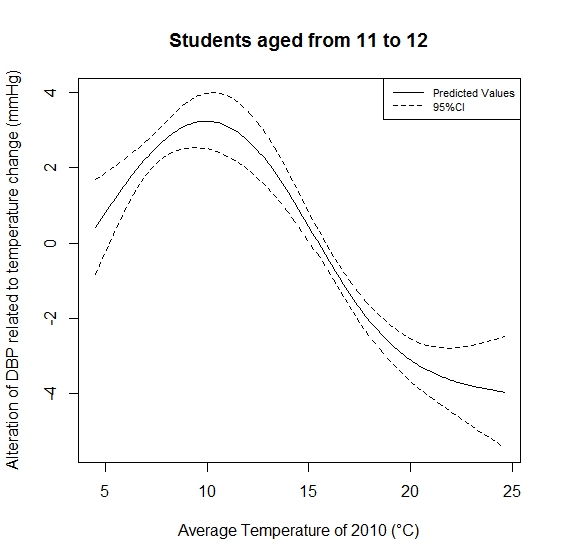


**Figure S3. The alteration of children’s SBP/DBP related to temperature difference**

**(11-12 years old children)**

(*Generalized additive model, adjusting for effect of school, gender, height, weight of each child, concentration of PM_10_, NO_2_ and SO_2_ of 2010, average relative humidity of 2010 and GDP per capital of 2010*)

**
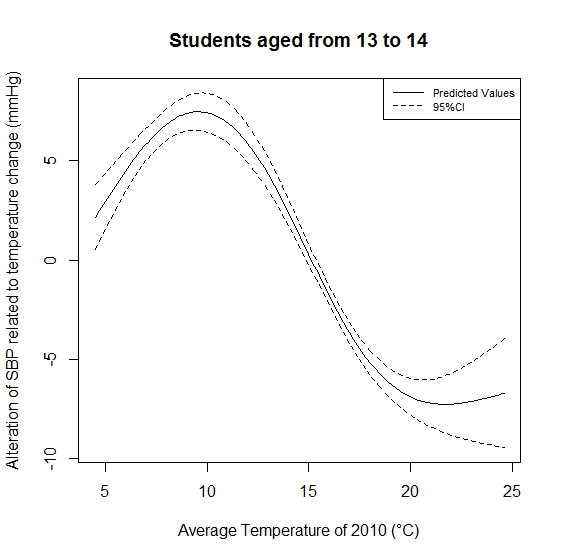
**
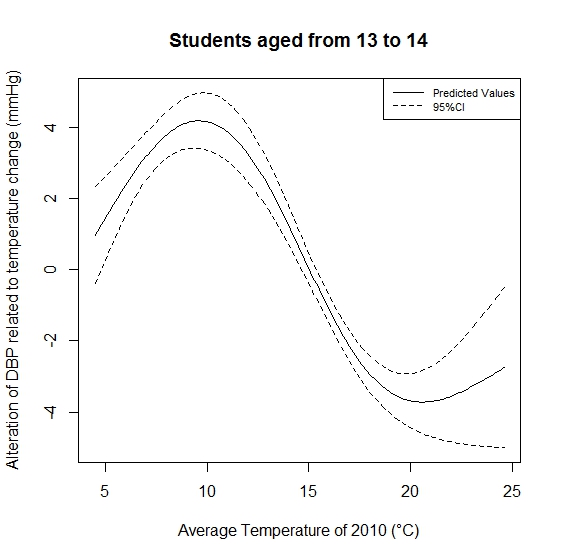


**Figure S4. The alteration of children’s SBP/DBP related to temperature difference**

**(13-14 years old children)**

(*Generalized additive model, adjusting for effect of school, gender, height, weight of each child, concentration of PM_10_, NO_2_ and SO_2_ of 2010, average relative humidity of 2010 and GDP per capital of 2010*)

**
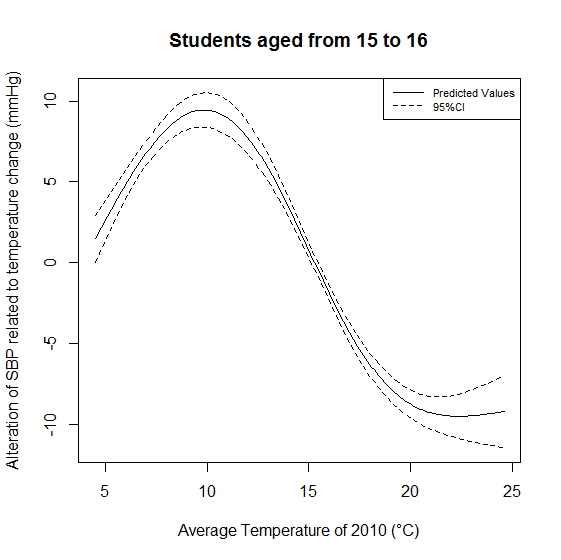
**
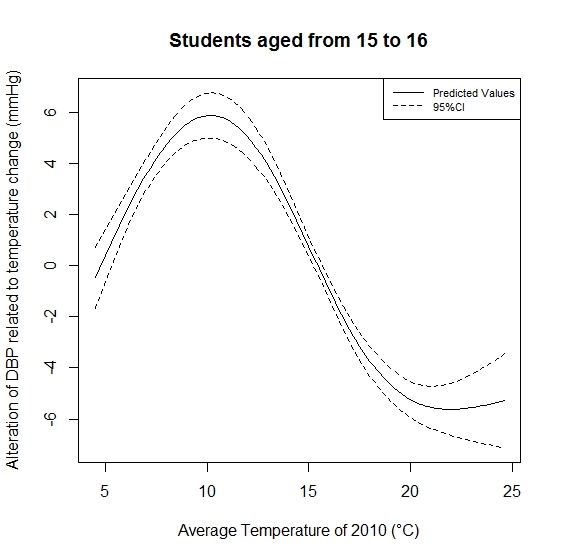


**Figure S5. The alteration of children’s SBP/DBP related to temperature difference**

**(15-16 years old children)**

(*Generalized additive model, adjusting for effect of school, gender, height, weight of each child, concentration of PM_10_, NO_2_ and SO_2_ of 2010, average relative humidity of 2010 and GDP per capital of 2010*)

**
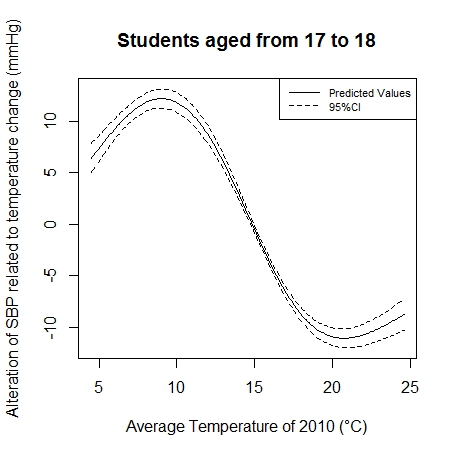
**
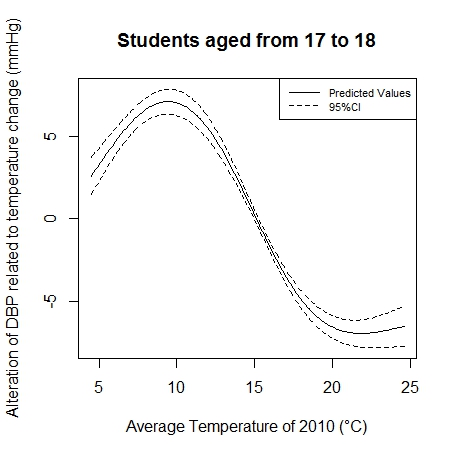


**Figure S6. The alteration of children’s SBP/DBP related to temperature difference**

**(17-18 years old children)**

(*Generalized additive model, adjusting for effect of school, gender, height, weight of each child, concentration of PM_10_, NO_2_ and SO_2_ of 2010, average relative humidity of 2010 and GDP per capital of 2010*)

**
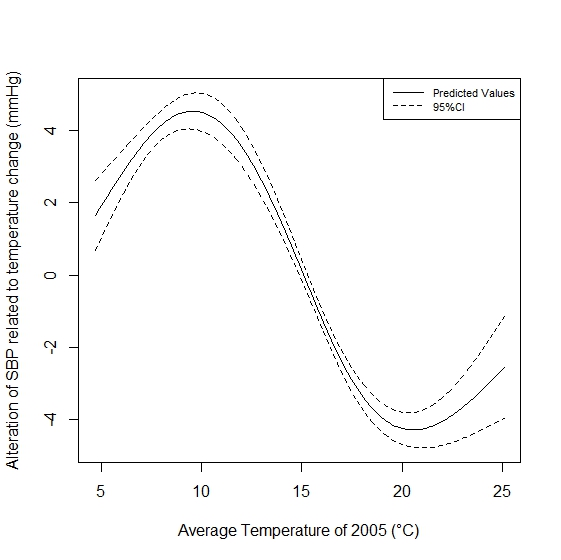
**
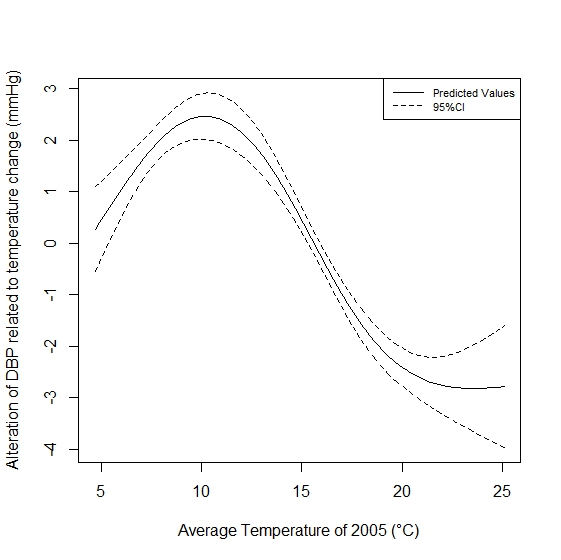


**Figure S7. The alteration of children’s SBP/DBP related to temperature difference** (*Generalized additive model, adjusting for effect of school, age, gender, height, weight of each child, concentration of PM_10_, NO_2_ and SO_2_ of 2010, average relative humidity of 2010 and GDP per capital of 2010, using annual average temperature of 2005*)

**
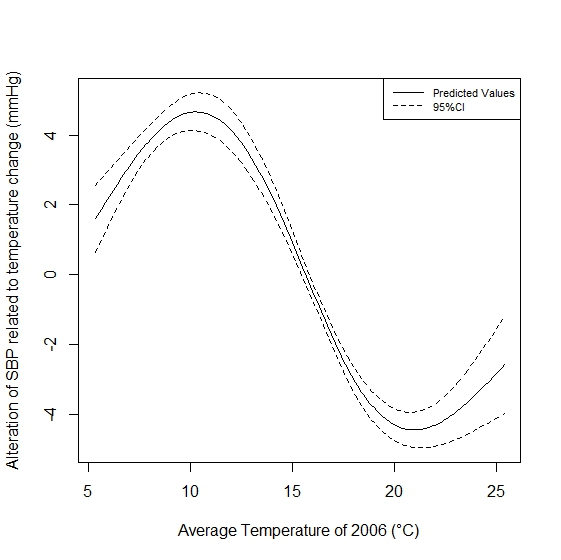
**
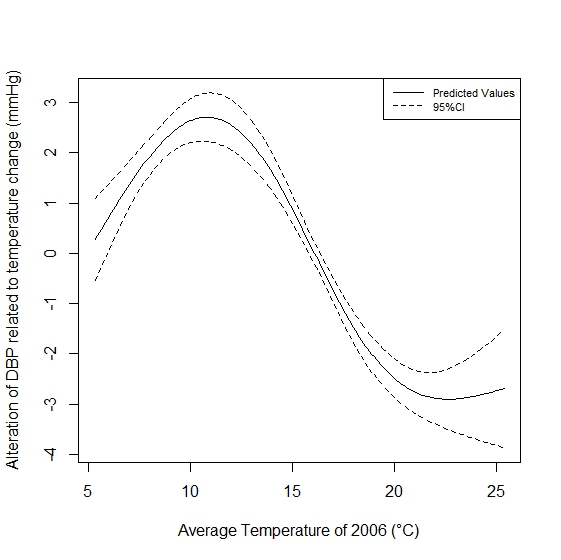


**Figure S8. The alteration of children’s SBP/DBP related to temperature difference** (*Generalized additive model, adjusting for effect of school, age, gender, height, weight of each child, concentration of PM_10_, NO_2_ and SO_2_ of 2010, average relative humidity of 2010 and GDP per capital of 2010, using annual average temperature of 2006*)

**
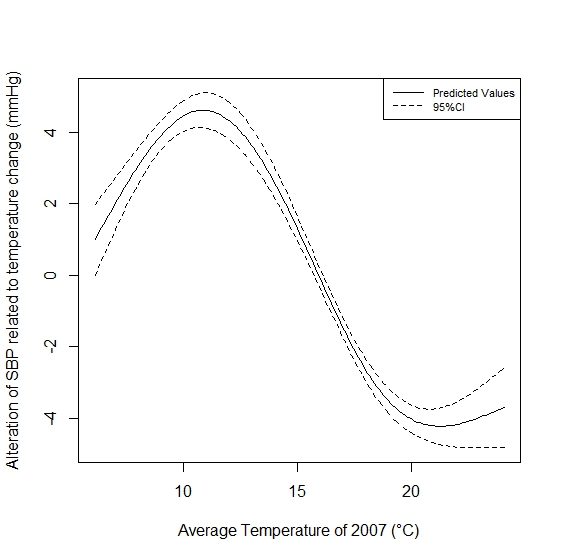
**
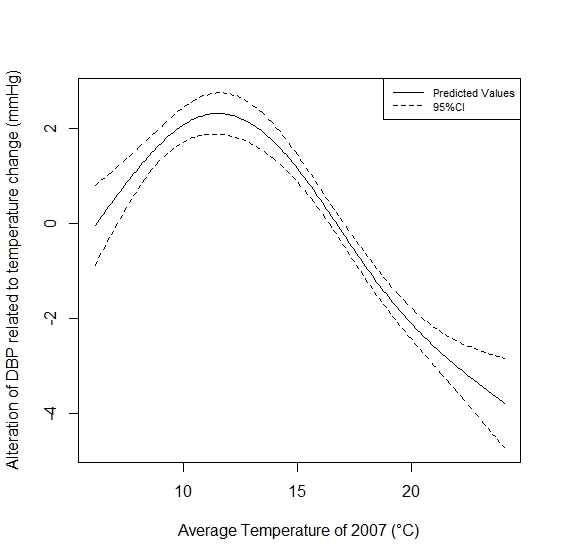


**Figure S9. The alteration of children’s SBP/DBP related to temperature difference** (*Generalized additive model, adjusting for effect of school, age, gender, height, weight of each child, concentration of PM_10_, NO_2_ and SO_2_ of 2010, average relative humidity of 2010 and GDP per capital of 2010, using annual average temperature of 2007*)

**
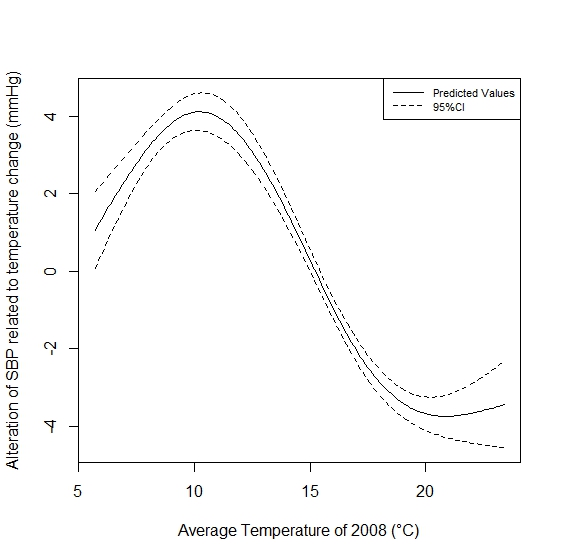
**
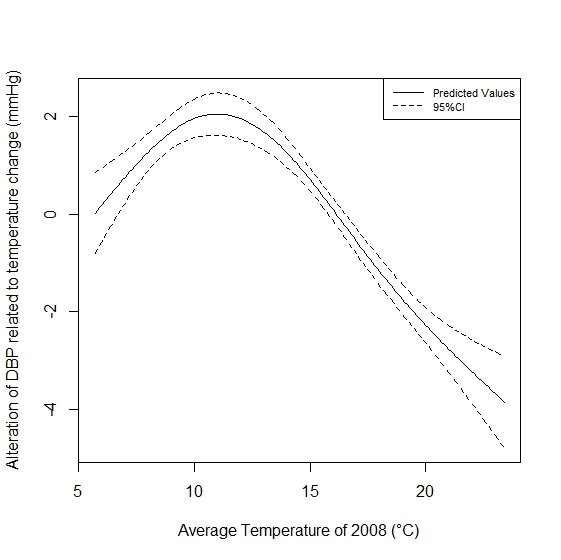


**Figure S10. The alteration of children’s SBP/DBP related to temperature difference** (*Generalized additive model, adjusting for effect of school, age, gender, height, weight of each child, concentration of PM_10_, NO_2_ and SO_2_ of 2010, average relative humidity of 2010 and GDP per capital of 2010, using annual average temperature of 2008*)

**
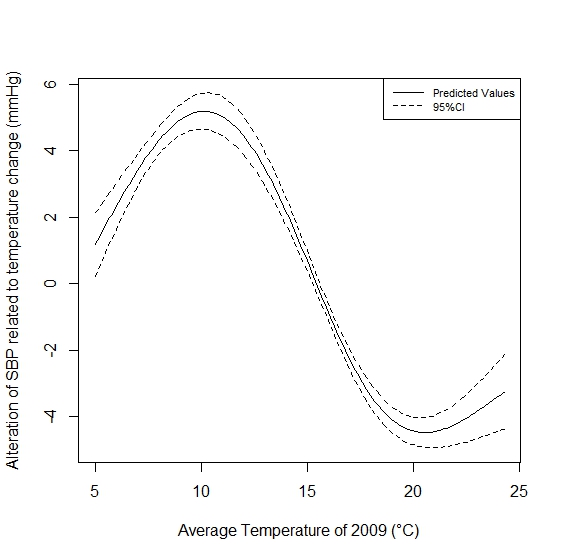
**
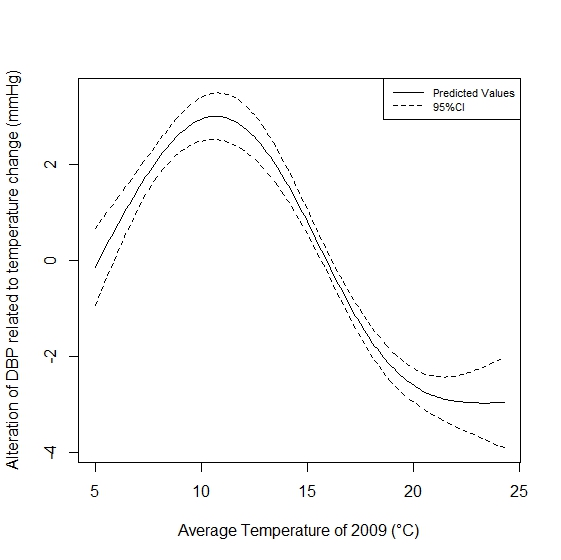


**Figure S11. The alteration of children’s SBP/DBP related to temperature difference** (*Generalized additive model, adjusting for effect of school, age, gender, height, weight of each child, concentration of PM_10_, NO_2_ and SO_2_ of 2010, average relative humidity of 2010 and GDP per capital of 2010, using annual average temperature of 2009*)

**
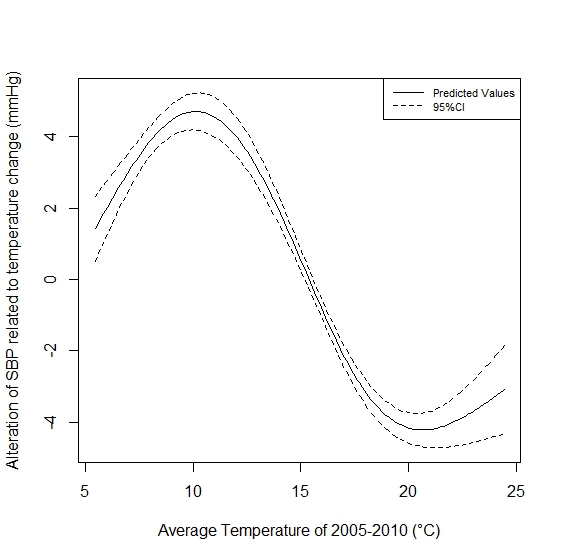
**
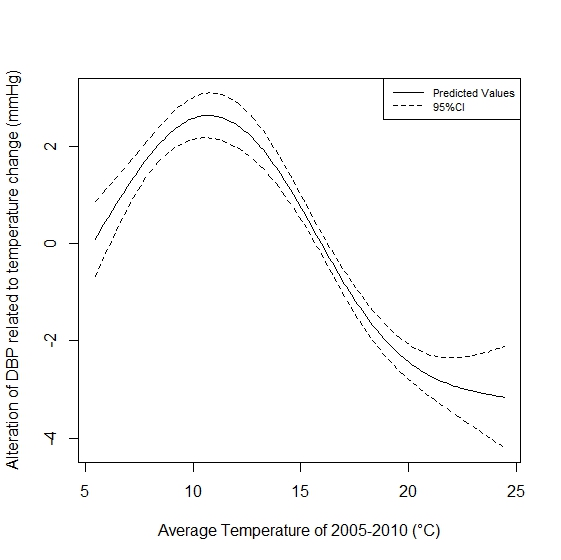


**Figure S12. The alteration of children’s SBP/DBP related to temperature difference** *(Generalized additive model, adjusting for effect of school, age, gender, height, weight of each child, concentration of PM_10_, NO_2_ and SO_2_ of 2010, average relative humidity of 2010 and GDP per capital of 2010, using mean annual average temperature from 2005 to 2010)*
